# Colonoscopy is mandatory after *Streptococcus bovis *endocarditis: a lesson still not learned. Case report

**DOI:** 10.1186/1477-7819-6-49

**Published:** 2008-05-12

**Authors:** Alberta Ferrari, Ivan Botrugno, Elisa Bombelli, Tommaso Dominioni, Emma Cavazzi, Paolo Dionigi

**Affiliations:** 1 Department of Surgery, University of Pavia, Istituto di Chirurgia Epatopancreatica, Fondazione IRCCS Policlinico San Matteo, Pavia, Italy

## Abstract

**Background:**

Even though the relationship between certain bacterial infections and neoplastic lesions of the colon is well-recognized, this knowledge has not been sufficiently translated into routine practice yet.

**Case presentation:**

We describe the case of a 51-year-old man who was admitted to our Surgical Department due to rectal bleeding and abdominal pain. Preoperative colonoscopy, staging exams and subsequent surgery demonstrated a stenotic adenocarcinoma of the sigmoid colon, invading the left urinary tract and the homolateral bladder wall, with regional lymph nodes involvement and massive bilobar liver metastases (T4N1M1). After Hartmann's rectosigmoidectomy and despite systemic chemotherapy, a rapid progression occurred and the patient survived for only 5 months after diagnosis. Five years before detecting this advanced colonic cancer, the patient underwent aortic valve replacement due to a severe *Streptococcus bovis *endocarditis. Subsequent to this infection he never underwent a colonoscopy until overt intestinal symptoms appeared.

**Conclusion:**

As this case illustrates, in the unusual setting of a *Streptococcus bovis *infection, it is necessary to timely and carefully rule out occult colon cancer and other malignancies during hospitalization and, if a tumor is not found, to schedule endoscopic follow-up. Rigorous application of these recommendations in the case described would have likely led to an earlier diagnosis of cancer and maybe saved the patient's life.

## Background

A well-recognized relationship has been established between unusual bacterial infections and neoplastic lesions of the colon. Although several bacteria have been reported in association with colonic cancer, the strongest and best documented relationship focuses on *Streptococcus bovis *[1,2]. *Streptococcus bovis *is classified as a non-enterococcal Streptococcus in Lancefield's group D and it is the pathogen agent of several types of infection including bacteremia, septicemia and endocarditis, but also unusual presentations such as endophthalmitis [3], soft tissue abscess [4], septic arthritis [5] and others. All types of *Streptococcus bovis *infection have been related to the presence of a gastrointestinal neoplasia, which in most cases is colonic adenoma or carcinoma.

Although there is agreement in the literature that this relationship has important clinical implications, their relevance hasn't yet been widely received. It has been suggested that the presence of *Streptococcus bovis *infection mandates complete gastrointestinal screening and, if negative, endoscopic follow-up [6]. Nevertheless, we report the case of a patient who was diagnosed with a very advanced colonic cancer five years after a severe *Streptococcus bovis *endocarditis. By reviewing the literature we discuss the failure in this patient's case to diagnose cancer earlier, underlining the need for more awareness about *Streptococcus bovis *infection and the risk of occult colonic tumor.

## Case Presentation

On January 2001, a 46 year-old male patient was admitted to hospital with intermittent low-grade fevers of unknown origin and severe asthenia that he had been experiencing for a month. His family history showed only one case of neoplastic disease among parents, 2 brothers and 5 sisters (his father died at 73 years due to stomach cancer). The patient was a hard smoker and his personal pathologic anamnesis didn't show any relevant disease other than traumatic bone fractures. The physical examination revealed good conditions except for the presence of fever and weakness. Lungs were clear but cardiac beats auscultation demonstrated a grade 2/6 systolic murmur. Laboratory examinations showed a normal complete blood count (white blood cells count: 8.8 × 10^9^/l with 74% polymorphonuclear leukocytes, hemoglobin: 12.6 g/dl), although a mild decreasing of medium red cells volume due to low blood iron (39 μg/dl) was found. Glucose level, hepatic and kidney function were also normal, while inflammatory tests resulted increased: C-Reactive Protein 8.1 mg/dl (normal 0.0–0.8 mg/dl), alpha-1-globulin 273 mg/dl (normal 33–88 mg/dl), erytrosedimentation rate test 43 mm/h (normal 0–10 mm/h). Tumor markers including CEA and Ca 19-9 were also evaluated and resulted not increased and fecal occult blood test was negative. X-ray examination of the chest was normal and ECG showed regular sinus rhythm and biphasic T waves. On the 2^nd ^hospitalization day an echocardiography was performed, demonstrating a small aortic valve vegetation associated with moderate regurgitation. These findings led to the diagnosis of infectious endocarditis and the patient was transferred to the Infectious Disease department of our hospital. A broad spectrum antibiotic therapy with ampicillin and gentamicin was empirically started and it continued since, after a few days, blood cultures demonstrated the growth of *Streptococcus bovis *sensitive to that antibiotic therapy. On the 21^st ^hospitalization day and after 3 weeks of antibiotic treatment the echocardiography still demonstrated two moving vegetations (the largest one measuring 23 mm in maximum diameter with surface area of 0.8 cm^2^) of the aortic valve adhering to the non coronary and coronary right cusps, associated with moderate regurgitation and mild pulmonary hypertension. Furthermore, since high intermittent fever reappeared, antibiotic treatment was empirically switched to vancomycin. Since this case of *Streptococcus bovis *endocarditis was considered to be at high risk of embolism, the patient was transferred to the Cardiosurgery department and on 32^nd ^hospitalization day he underwent the replacement of the aortic valve with mechanical prosthesis. The postoperative course was uneventful; vancomycin treatment was switched to teicoplanin on the basis of antimicrobial susceptibility and finally the patient was discharged. The one month follow-up after cardiosurgery showed the patient to be in good clinical conditions.

No further complications occurred for more than five years after the successfully treated *Streptococcus bovis *endocarditis and the patient underwent no clinical check-ups or diagnostic evaluations.

**Figure 1:**
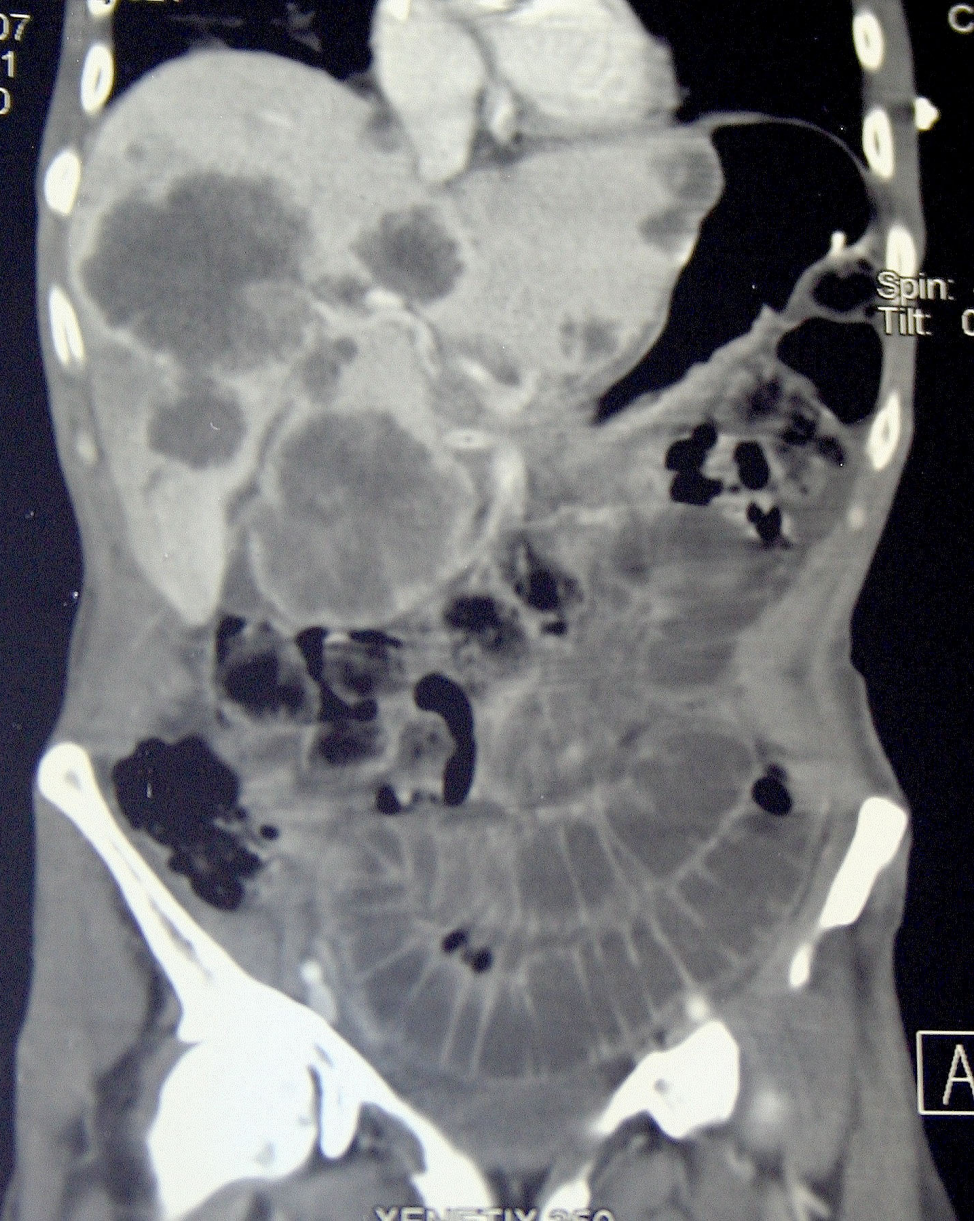
Abdominal CT scan performed before surgery shows both mechanical bowel obstruction and diffuse liver focal lesions due to advanced metastatic disease.

On November 2006, the same patient went to his family doctor complaining of 15% weight loss in the last three months, along with asthenia and constipation. Blood exams revealed hypocromic microcitic anemia (haemoglobin 8.8 g/dl), high levels of carcinoembryonic antigen (CEA: 2221 ng/ml) and fecal occult blood test was positive. Abdominal pain and rectal bleeding occurred a few days after those exams and the patient was admitted to our Surgery department. A colonoscopy was performed revealing sigmoid colon stenosis: the exploration of the remaining tracts of the colon was not possible due to the severe obstruction. Histological examination of the biopsies demonstrated a sigmoid colon adenocarcinoma. In addition to the bowel mechanical obstruction, both abdominal ultrasound and CT scan revealed the presence of several focal liver lesions with widespread bilobar diffusion (figure 1). Laparotomic surgery was then performed: the intraoperative findings confirmed advanced sigmoid colon tumor with pelvic diffusion, direct invasion of the left bladder wall and of the left urinary tract and multiple bilobar liver metastases. A palliative Hartmann's resection of the upper rectum and sigma with left colostomy and a biopsy of hepatic lesions were performed. The postoperative course was uneventful.

The definitive histological examination of the resected sigmoid colon confirmed the presence of a moderately differentiated (G2) adenocarcinoma of the large bowel infiltrating the whole thickness of the wall and perivisceral tissues, with a secondary nodule on the serous surface; it had an infiltrative growth pattern with lymphatic invasion and with a poor peritumoral lymphocytic reaction. One out of 23 regional lymph nodes was involved by the tumor, and hepatic biopsy confirmed the clinical evidence of widespread liver metastatic diffusion. The final pathological stage was a modified Dukes D (T4N1M1).

Despite an aggressive polichemotherapy regimen started on December 2006, the tumor showed a dramatically rapid progression. On April 2007, the patient underwent surgery again, due to intestinal occlusion; a preoperative CT scan demonstrated massive pelvic recurrence and right lung neoplastic lymphangitis. The laparotomic surgery confirmed the pelvic mass with diffuse peritoneal carcinosis, so a palliative enteric anastomosis by-passing the main site of occlusion was performed. The immediate postoperative course was characterized by persistent shock and multiorgan failure not responsive to intensive care unit support and twelve hours after surgery the patient died. Patient survival after colonic cancer diagnosis was 5 months only.

## Discussion

The occurring of a bacterial endocarditis together with colonic carcinoma was first reported in 1951 [7], however it was only in 1977 that *Streptococcus bovis *was recognized by Klein et al. as the pathogen agent specifically related to the presence of a colonic cancer [1]. Although many authors have reported a relationship between this kind of tumor and many bacterial strains, the strongest and best documented association remains the one between colonic cancer and *Streptococcus bovis *infection.

Many other case reports and two prospective studies in the literature confirmed the hypothesis that the development of *Streptococcus bovis *infection could represent the first sign of a colonic cancer. The first series was reported in 1979 by Klein et al. [8]: by a complete gastrointestinal evaluation of 15 patients with *Streptococcus bovis *septicemia, 13 cases (86,6%) of tumors were found. In particular, 11 patients had colonic diseases including 2 adenocarcinomas, 6 microcarcinomas (detected in 5 villous adenomas and 1 adenomatous polyp) and 3 benign adenomatous polyps; 2 other patients were affected by esophageal carcinoma. From this study an important lesson was learned for the first time: in most cases of *Streptococcus bovis *infection a concomitant colorectal cancer can be expected and this evidence mandates endoscopic examination. Moreover, the presence of an upper gastrointestinal tract malignancy must also be considered. The second prospective study in the literature reported by Wilson et al. in 1981 [9] confirmed the high (62%) prevalence of colonic disease in 21 patients affected by *Streptococcus bovis *endocarditis, even if in this series most patients had benign pathologies (inflammatory bowel disease, diverticula, polyps or villous adenoma) and only 5% were affected by colonic cancer.

The pathogenesis of the association between *Streptococcus bovis *infection and colonic disease has been investigated by several studies, however it is still not clear. *Sreptococcus bovis *is a normal inhabitant of the human gastrointestinal tract, as demonstrated by the fact that it can be found in the fecal specimens of about 5–16% of healthy population. An increased percentage of up to 56% has been reported in the case of inflammatory bowel disease or colonic cancer [1], but this finding has not been confirmed in more recent studies [10]. The hypothesis that ulceration of the neoplastic lesion would directly open a pathway for the bacteria to enter the bloodstream does not explain the case of association between *Streptococcus bovis *and non ulcerated colonic polyps or adenoma. It seems more likely that a bacterial translocation without the need for mucosal disruption may occur due to vascular changes related to several gastrointestinal diseases [11]. A further association between *Streptococcus bovis *bacteremia and liver disease has been reported, thus suggesting that an altered hepatic function (secretion of bile salts, production of immunoglobuline) may play a role in the alteration of colonic flora and/or bacterial translocation [12,13]. A recent study suggests the intriguing hypothesis that the majority of patients affected by colonic cancer develop a silent infection, although it only becomes apparent when immune system disorders or cardiac valve lesions occur. Identification of tumor-associated *Streptococcus bovis *silent infectionthrough profiling the humoral immune response represents a promising potential means for prevention and early diagnosis of colonic cancer [14]. Finally, a direct carcinogenetic role of *Streptococcus bovis *is possible because of its demonstrated capability in a rat model to promote the pre-neoplastic colonic lesions progression [11].

Although the knowledge about the true pathophysiologic relationship between *Streptococcus bovis *infection and gastrointestinal neoplasia needs further studies, it is already well-recognized that a strong association does exist and has important clinical implications. Since early reports [1,2,15] until now it has been demonstrated that endoscopic screening is able to detect occult benign, pre-malignant and cancerous diseases of the colon in most patients with *Streptococcus bovis *infection [12,16]. As recently reported by Gold et al. this finding ranges from 6% to 71% in the reviewed literature [17]. Furthermore, the same authors also underline the previously underestimated association between *Streptococcus bovis *infection and extracolonic and even extraintestinal malignancies.

On the basis of these data, in the last decades, several authors have advocated the need for an appropriate endoscopic screening for polyps and malignancies even in asympthomatic patients when a *Streptococcus bovis *infection is recognized [1-6,15-18]. Notably, the *Streptococcus bovis *group of bacteria has been recently reclassified based on DNA-DNA hybridisations and phylogenetic analyses of 16S RNA gene sequences [19]; on this basis biotypes I and II.2 were renamed *Streptococcus gallolyticus *(subsp. *gallolyticus *and subsp. *pasteurianus*, respectively). Since these changes in nomenclature may represent a pitfall in recognizing an underlying occult colon tumor [20], we recommend doctors to be alerted that a diagnosis of *Streptococcus gallolyticus *infection has the same clinical implications of *Streptococcus bovis *[21]. Furthermore, *Streptococcus gallolyticus *subsp. *gallolyticus *is the new name of *Streptococcus bovis *biotype I, which has been more commonly associated with occult cancer [22], so that the need for endoscopic screening is even stronger in this case.

Even though it is already well-recognized that the clinical setting of a *Streptococcus bovis *(or *gallolyticus*) infection mandates a diagnostic work-up to reveal an occult neoplasia, it seems that awareness among physicians who take care of these patients is still poor, not only due to the pitfall of nomenclature. Gold et al. have warned about the underutilization of colonoscopy in their patient population with *Streptococcus bovis *bacteremia [17] and Wentling et al. have recently suggested that diagnostic assessment should be scheduled before hospital discharge [6].

Our experience sheds light on the importance of performing a complete diagnostic assessment to rule out an occult colon or even extracolon cancer *during *inpatient treatment, avoiding focusing only on infectious disease treatment.

Notably, data collected from the published series demonstrate that performing screening colonoscopy after *Streptococcus bovis *infection allows the detection of colonic neoplasia in early or pre-cancerous stages in most cases [8,16,17]. This finding has been recently supported by a study on bacterium antigen profiles, showing that infection occurs early during carcinogenesis [14]. Moreover, it has been suggested that a negative diagnostic assessment at the time of infection is not enough, because a colonic polyp or cancer may develop several years after *Streptococcus bovis *infection [18,23]. While waiting for new technologies for colonic cancer screening, colonoscopy still remains the most effective tool to follow-up such patients at risk of colon cancer. The frequency of endoscopic examination in such patients has not been established yet, however in our opinion, the demonstrated high risk of developing a colon neoplasia would justify an annual colonoscopic screening.

The presence in our patient of a sigmoid adenoma or cancer at the time of *Streptococcus bovis *endocarditis is uncertain because the lesion had not been investigated. However, even if the overall impact of endoscopic examination and follow-up on survival in patients who have been affected by *Streptococcus bovis *infection is unknown, in the case here reported we are legitimate to suppose that an annual surveillance would have led to an earlier diagnosis and potentially curative treatment, thus saving the patient's life.

## Conclusion

In the unusual setting of a *Streptococcus bovis *infection, this case stresses the need to timely and carefully rule out occult colon cancer and other malignancies during hospitalization and, if a tumor is not found, to schedule an annual endoscopic follow-up.

## Competing interests

The authors declare that they have no competing interests.

## Authors' contributions

AF: principle investigator who prepared, organized, wrote, and edited all aspects of the manuscript. IB: surgical oncologist involved in identification of relationship between colon cancer and previous *Streptococcus bovis *infection. EB: involved in clinical management and evaluation of the literature. TD: supported the work of principle investigator in preparing the manuscript. EC: supported the work of principle investigator in writing and editing the manuscript. PD: he read, edited, and approved the final version of the manuscript. All authors read and approved the final version of the manuscript.
